# Utilizing Learn-to-Rank Systems for More Effective Diagnosis in Rural Family Medicine

**DOI:** 10.7759/cureus.47219

**Published:** 2023-10-17

**Authors:** Ryuichi Ohta, Chiaki Sano

**Affiliations:** 1 Community Care, Unnan City Hospital, Unnan, JPN; 2 Community Medicine Management, Shimane University Faculty of Medicine, Izumo, JPN

**Keywords:** vuca, healthcare innovation, cultural resonance, medical technology integration, family medicine, rural healthcare, disease diagnosis, clinical decision support systems (cdss), learn-to-rank approach

## Abstract

This editorial investigates the development and efficacy of Japanese learn-to-rank approach systems in family medicine, emphasizing their establishment by Dr. Keijiro Torigoe and their significance in rural community hospitals. Initiated in 1977, Dr. Torigoe’s innovative system integrated international medical knowledge with technology, yielding a comprehensive database of 7,000 registered diseases. These learn-to-rank approaches, notably the listwise method, address technological gaps in extracting data on differential diseases and enhance the predictive performance of clinical decision support systems, offering a holistic, culturally resonant healthcare approach. They are especially vital in rural medicine, aiding in managing the volatility, uncertainty, complexity, and ambiguity prevalent among older patients, streamlining diagnoses, and improving healthcare delivery in resource-constrained settings. In conclusion, integrating Japanese learn-to-rank approach systems is pivotal in revolutionizing disease diagnosis, catering to diverse rural health needs, and fostering sustainability in rural healthcare systems. By harmonizing medical insights with innovation, they demonstrate the potential for a comprehensive and contextually relevant approach to healthcare in Japan.

## Editorial

Background

Family physicians stand at the forefront of healthcare, navigating the challenging waters of multiple diseases that affect individuals throughout their lifespan [1]. Approaching these diverse diseases is essential because it lays the foundation for comprehensive care and fosters the well-being of communities [1]. Effective diagnosis is the cornerstone of this approach, with a critical need for sustainable methods that accommodate the ever-evolving nature of diseases and patient needs. The effective diagnosis can be supported by diagnosis-supporting systems.

Adopting diagnosis-supporting systems has become increasingly prevalent in the modern medical landscape [2]. These innovative systems leverage advancements in technology and medical knowledge to aid physicians in making accurate and timely diagnoses. These embody a synthesis of medical expertise and technological innovations aimed at enhancing the overall efficacy of the diagnostic process.

However, the effectiveness of these diagnosis-supporting systems is not universal, as they are closely intertwined with the cultural and contextual nuances of different regions and populations [3]. The need for culturally sensitive and contextually appropriate systems is paramount, ensuring they are adaptable and resonate with the diverse patient demographics encountered by family physicians.

For the effective usage of diagnosis-supporting systems, this editorial delves into Japanese diagnosis-supporting systems within this context respecting the culture and environments of rural family medicine, and highlighting a rural family physician's continuous establishment and enhancement. In collaboration with various engineers, physicians play a pivotal role in tailoring these systems to meet the unique needs and expectations of the local population. By exploring these systems, this editorial highlights the significance of context and culture in developing and utilizing diagnosis-supporting tools, thereby contributing to a broader discourse on sustainable and effective disease diagnosis and management.

The significance of Japanese learn-to-rank approaches for the diagnosis of diseases by physicians

Integrating Japanese learn-to-rank approaches into the diagnostic process significantly aligns technological advancements with physicians' nuanced iterative strategies for differential diagnosis [3]. These approaches, precisely the listwise method, mirror the physicians' workflow of recalling, refining, and ranking multiple differential diseases, thereby fostering a symbiotic relationship between machine-learning frameworks and medical expertise.

A pivotal aspect of this integration is its potential to address existing technological gaps [3]. Current technologies are proficient in text-mining information related to confirmed diseases but fall short in extracting and converting data on multiple differential diseases from a vast body of medical literature. Japanese learn-to-rank approaches could bridge this gap, particularly the listwise approach, ensuring a more comprehensive and informed diagnostic process.

Moreover, the significance of these approaches is underscored by the urgent need to enhance the predictive performance of clinical decision support systems (CDSS) [3]. By applying strict criteria focused on rare and difficult-to-diagnose cases, these approaches promise to cater to real-world challenges encountered by internists and general practitioners. Instead of relying on unvalidated real-world data, the meticulous derivation of case data from various medical literature further accentuates the reliability and applicability of learn-to-rank approaches in the Japanese medical context.

Furthermore, by addressing the limitations of the conventional CDSS, which predominantly employ a pointwise approach and often omit crucial information on differential diseases, the Japanese learn-to-rank approaches aim to create a more holistic and affinitive diagnostic tool. This enhances the accuracy and depth of the diagnostic process and aligns more closely with the differential diagnosis processes of experienced physicians, ensuring a more patient-centered and culturally resonant approach in healthcare [4].

The significance of integrating Japanese learn-to-rank approaches lies in their potential to revolutionize the diagnosis of diseases by harmonizing technological innovation with medical insights, addressing the limitations of existing systems, and ensuring a more comprehensive, accurate, and contextually relevant approach to healthcare in Japan.

How to establish the Japanese learn-to-rank approach system

In 1977, a groundbreaking development emerged in Japan, initiating the Japanese learn-to-rank approach system of supporting clinical diagnosis. This innovative model was designed to structure and prioritize medical information, primarily focusing on patients’ diagnosis and management. The linchpin of this endeavor was Keijiro Torigoe, a family physician based in Okayama, Japan [4]. Torigoe's remarkable methodology involved restoring diseases based on a comprehensive study of case reports published in international journals and integrating global knowledge into a localized system [4].

Torigoe, committed to refining and enhancing the capabilities of this system, fostered collaboration with system engineers and amalgamated medical expertise through technological innovation. This synergy has resulted in continuous revisions and optimizations, leading to a significantly expanded and enriched database. The system burgeoned, and registered diseases exponentially escalated to approximately 7,000. This expansion reflects the breadth of the medical conditions addressed, the depth of understanding, and the capacity for nuanced, detailed analysis within the system [3].

This introduction lays the foundation for exploring the intricacies and impacts of the Japanese learn-to-rank approach system, focusing on its inception, development, and the pivotal role of Keijiro Torigoe in amalgamating international knowledge with technological advancements to create a comprehensive disease database. Exploring this innovative model provides insights into integrating medical knowledge and technology for optimized healthcare solutions.

The effectiveness of the learn-to-rank approach system in rural community hospitals

Family physicians, particularly those practicing in rural areas, are increasingly finding it necessary to familiarize themselves with learn-to-rank approaches when diagnosing older patients who present with multiple symptoms. Learn-to-rank is a class of machine learning techniques that sorts items by relevance or importance, which in family medicine can aid in prioritizing potential diagnoses or interventions [5]. In rural family medicine, volatility, uncertainty, complexity, and ambiguity (VUCA) among older patients presents a considerable challenge, necessitating an enhanced approach and education among rural family physicians to effectively address a diverse range of health issues [5].

The learn-to-rank approach system is especially suitable for addressing the prevalent issue of VUCA. By integrating this system into the diagnostic process, physicians can swiftly and accurately assess and prioritize older patients' symptoms, thereby tailoring the most effective treatment plan. This is particularly beneficial in rural settings, where healthcare resources are often limited, and the range of medical expertise may be constrained [3,5]. Introducing learn-to-rank approach systems can bridge this gap, providing invaluable support to rural family physicians in diagnosing and managing various health conditions.

Furthermore, this innovative diagnostic support system will be instrumental in overcoming the inherent challenges rural healthcare providers face. The scarcity of healthcare resources and the diverse health needs of older patients necessitate adopting advanced technological solutions such as learn-to-rank approach systems [5]. These systems can significantly alleviate the burden on healthcare professionals working in resource-limited settings by facilitating streamlined and effective diagnostic processes. This enhances the accuracy and efficiency of diagnoses and improves healthcare delivery in rural areas.

Conclusion

Practical implementation of learn-to-rank approach systems is pivotal for mitigating the burden on healthcare professionals. In rural family medicine, where resources are scarce and patient populations are diverse, prompt and accurate diagnosis using these systems is crucial. By supporting physicians in their diagnostic processes, these systems foster a more sustainable rural healthcare environment, allowing better management of healthcare resources and ensuring that older patients' diverse and complex needs are adequately met. By adopting and integrating learn-to-rank approach systems into the diagnostic workflow, rural family physicians can significantly enhance the quality of care provided to patients, thereby contributing to rural healthcare systems' long-term sustainability and effectiveness.
